# HARNESSING THE POWER OF NETWORKS: PARTNERSHIPS TO PROMOTE HEALTH AND WELL-BEING FOR FAMILY CAREGIVERS

**DOI:** 10.1093/geroni/igz038.1981

**Published:** 2019-11-08

**Authors:** Heather Young

**Affiliations:** Betty Irene Moore School of Nursing - UC Davis, Sacramento, California, United States

## Abstract

This paper will address social and professional networks that support family caregivers who are providing care to older adults. Family caregiving is commonly a long-term commitment that can include functional, health care, social, financial and emotional support. This role can have a major impact on the health, well-being and economic security of the family caregiver, particularly for those who are socially isolated. This paper will highlight the nature of caregiving demands on individuals and families, particularly those providing intense and complex care, with a focus on cultural diversity. Health care professionals encounter family caregivers during routine care and at times of crisis and have an opportunity to enhance support and preparation. This paper will discuss effective community-level and health system partnerships and strategies that promote health and well-being for family caregivers, examine the potential for scaling these approaches, and suggest priorities for education, practice and future research.

